# News from Federal Health Reporting

**DOI:** 10.17886/RKI-GBE-2018-054

**Published:** 2018-05-16

**Authors:** Franziska Prütz

**Affiliations:** Robert Koch Institute, Berlin, Department of Epidemiology and Health Monitoring

Federal Health Reporting (GBE) continuously provides current data and information on the German population’s health and health care services utilisation. This article provides information on present GBE focuses: the Journal of Health Monitoring, as well as three projects, which belong to the field of Gender and Health – the report on women’s health in Germany, and the Frauen 5.0 and AdvanceGender projects.

Since September 2016, GBE has been publishing the Journal of Health Monitoring. Available online in German and English, this new format is open access, barrier-free and linked to the Federal Health Reporting information system (www.gbe-bund.de) and aims i. a. to improve the visibility of Federal Health Reporting. Work in 2017 focused on a basic evaluation of the GEDA 2014/2015-EHIS health survey. In 2018, the focus is on KiGGS Wave 2.

Following the first report on women’s health in Germany (Frauengesundheitsbericht), which was published in 2001, a new report is currently being developed and will highlight important aspects related to the health of women in Germany. In its first part, based on GBE data, the report will provide a general overview of the health of women in Germany [1]. Subsequently, part two will consist of various focus chapters touching on important aspects of women’s health, such as sexual and reproductive health, or deal with the health of particular groups of women (for example women with a migration background, women with disabilities, and elderly women). The report will include comparative gender analyses, as well as analyses regarding the health of specific sub-groups (for example stratified by age or socioeconomic status). Beyond merely discussing prevalences, the report will provide an interpretation of data and discuss explanation approaches.

AdvanceGender (a joint research project between the Charité in Berlin, Bremen University and the RKI), in turn, focuses on more fundamental questions of gender equity in health reporting. This should lead to a best practice model for gender sensitive health reporting across the entire research process (from data collection and analysis to health reporting).

Frauen 5.0 (conducted jointly by the Charité Berlin and the RKI) focuses on a very specific field of women’s health: analysing the health and health care utilisation of women aged over 49 years in rural areas, in particular regarding coverage of gynaecology and general medical services and developing proposals for new models of health care.
